# Ketone bodies mediate alterations in brain energy metabolism and biomarkers of Alzheimer’s disease

**DOI:** 10.3389/fnins.2023.1297984

**Published:** 2023-11-16

**Authors:** Matin Ramezani, Malika Fernando, Shaun Eslick, Prita R. Asih, Sina Shadfar, Ekanayaka M. S. Bandara, Heidi Hillebrandt, Silochna Meghwar, Maryam Shahriari, Pratishtha Chatterjee, Rohith Thota, Cintia B. Dias, Manohar L. Garg, Ralph N. Martins

**Affiliations:** ^1^Macquarie Medical School, Faculty of Medicine, Health and Human Sciences, Macquarie University, Macquarie, NSW, Australia; ^2^Flinders Health and Medical Research Institute, College of Medicine and Public Health, Flinders University, Adelaide, SA, Australia; ^3^Motor Neuron Disease Research Centre, Macquarie Medical School, Faculty of Medicine, Health and Human Sciences, Macquarie University, Sydney, NSW, Australia; ^4^School of Medical and Health Sciences, Edith Cowan University, Joondalup, WA, Australia

**Keywords:** ketogenic intervention, disease-modifying therapy, Alzheimer’s disease, circulating biomarkers, metabolic interaction, ketogenesis, brain energy fuel

## Abstract

Alzheimer’s disease (AD) is the most common form of dementia. AD is a progressive neurodegenerative disorder characterized by cognitive dysfunction, including learning and memory deficits, and behavioral changes. Neuropathology hallmarks of AD such as amyloid beta (Aβ) plaques and neurofibrillary tangles containing the neuron-specific protein tau is associated with changes in fluid biomarkers including Aβ, phosphorylated tau (p-tau)-181, p-tau 231, p-tau 217, glial fibrillary acidic protein (GFAP), and neurofilament light (NFL). Another pathological feature of AD is neural damage and hyperactivation of astrocytes, that can cause increased pro-inflammatory mediators and oxidative stress. In addition, reduced brain glucose metabolism and mitochondrial dysfunction appears up to 15 years before the onset of clinical AD symptoms. As glucose utilization is compromised in the brain of patients with AD, ketone bodies (KBs) may serve as an alternative source of energy. KBs are generated from the β-oxidation of fatty acids, which are enhanced following consumption of ketogenic diets with high fat, moderate protein, and low carbohydrate. KBs have been shown to cross the blood brain barrier to improve brain energy metabolism. This review comprehensively summarizes the current literature on how increasing KBs support brain energy metabolism. In addition, for the first time, this review discusses the effects of ketogenic diet on the putative AD biomarkers such as Aβ, tau (mainly p-tau 181), GFAP, and NFL, and discusses the role of KBs on neuroinflammation, oxidative stress, and mitochondrial metabolism.

## 1. Introduction

Alzheimer’s disease (AD) has emerged as one of the most severe health-threatening conditions in the 21st century (Rolandi et al., 2020). The incidence rate of AD is expected to rise with a disproportionate increase in low- and middle-income societies (Zhang et al., 2021). From 1990 to 2019, the prevalence and mortality rates of disease have doubled (Javaid et al., 2021). Currently, more than 50 million people worldwide are living with dementia, particularly AD accounting for an estimated 50–70% of all dementia cases (Zhang et al., 2021). This number is estimated to increase to 150 million by the end of 2050 (Patterson, 2018). As AD prevalence and mortality rates are increasing worldwide, it is crucial to advance our understanding of the disease (Patterson, 2018), which will considerably support the development of therapies (Cunnane et al., 2011).

Aging is the most prominent risk factor for developing AD with predominantly diagnosed as late-onset Alzheimer’s disease in people above 65 years old (Hoogmartens et al., 2021). More than 90% of AD is sporadic predominantly with a late onset, due to a combination of genetic variants (70%) such as APOE4 and environmental factors (30%), such as hormonal and molecular changes, diet and toxicological exposure (Dorszewska et al., 2016). However, early onset of AD can predominantly be diagnosed in patients with familial AD, due to mutations in the Amyloid Precursor Protein (APP), presenilin 1 (PSEN1) and presenilin 2 (PSEN2) genes (Piaceri et al., 2013; Hoogmartens et al., 2021).

Neuropathology of AD starts approximately 15–20 years prior to its clinical symptoms (Ashton et al., 2019). This preclinical phase in AD is a “symptom free” stage, and individuals are cognitively unimpaired with amyloid aggregation (Vermunt et al., 2022). The more progressive Aβ aggregation would be found in prodromal phase of AD referring to the mild cognitive impairment (MCI) with an impairment in at least one cognitive domain (Douglas and Scharre, 2019; Vermunt et al., 2022), and severe AD dementia or symptomatic AD is the late-stage AD characterized by a progressive functional impairments, and more definitive clinical symptoms (Douglas and Scharre, 2019).

Classical neuropathological hallmarks of AD, such as amyloid beta (Aβ) plaques and neurofibrillary tangles containing the neuron-specific protein tau has been reported in the preclinical phase of AD (Gunes et al., 2022). The most advanced AD biomarkers with a greater prognostic and diagnostic value that are changed in cerebrospinal fluid (CSF), and plasma include amyloid beta (Aβ), phosphorylated tau (p-tau), neurofilament light (NFL) and glial fibrillary acidic protein (GFAP) (Benedet et al., 2021; Chatterjee et al., 2023). Any neuropathological changes such as reactive astrogliosis (Salvadó et al., 2022) and disruption of the neural axonal cytoskeletal structure (Poff et al., 2021; Alberti et al., 2022) can change the level of these circulating biomarkers (Poff et al., 2021; Alberti et al., 2022), thereby making these biomarkers as the promising indicators for the early diagnosis of AD (Khoury and Ghossoub, 2019; Ausó et al., 2020; Klyucherev et al., 2022). Understanding these changes will proceed early diagnosis and therefore more effective disease-modifying approaches (Arvanitakis et al., 2019).

Furthermore, AD-affected brain shows glucose hypometabolism due to alleviated glucose uptake and utilization through different types of glucose transporters (GLUTs) (Szablewski, 2021). Predominantly, glucose uptake into the brain occurs using transporters GLUT1 and GLUT3 (Kyrtata et al., 2021). In AD brain, a reduced expression of GLUT1 carriers localized in brain microvasculature and astrocytes (Jurcovicova, 2014) as well as a decline in GLUT3 expressed in neurons have been indicated (Szablewski, 2021). In contrast, GLUT2 as an insulin-sensitive glucose transporter is highly expressed in AD pathology assumed to be due to astrogliosis (Kyrtata et al., 2021). These subsequently result in reduced ATP production from glucose metabolism by 50% that continues to decrease further with disease progression (Szablewski, 2021). Brain energy deficits arising from the aforementioned processes are further attributed to AD-related neuropathological changes (Poff et al., 2021; Alberti et al., 2022; Salvadó et al., 2022).

In the brain neurons produce the majority of ATP through the oxidative phosphorylation of ADP. However, glia cells are also responsible for ATP synthesis (Beard et al., 2022). Preferentially, astrocytes undergo glycolysis to synthesize lactate and pyruvate from glucose (Chamberlain and Sheng, 2019; Beard et al., 2022). Astrocytes due to having glycolytic enzymes are capable of using 80% of the glucose via glycolysis, while glycolytic enzymes are inhibited in the neurons (Chamberlain and Sheng, 2019). Albeit decreased glycolysis is associated with early cognitive impairment, contributing to AD progression (Goyal et al., 2023).

These abnormal glucose homeostasis in the brain (An et al., 2018) such as reduced uptake and utilization of brain glucose, perturbed glucose metabolism, reduced glycolysis and insulin and insulin-like growth factor-1 (IGF-1) resistance can cause a deficit in brain energy metabolism reported in AD brain (De La Monte, 2012; Szablewski, 2021). Glucose deficiency can subsequently result in reduced ATP production by 50% that continues to decrease further with disease progression (Hoyer, 1992; Szablewski, 2021). Brain energy deficits arising from the aforementioned processes are further attributed to AD-related neuropathological changes (Poff et al., 2021; Alberti et al., 2022; Salvadó et al., 2022). Glucose deficiency not only cause an energy crisis which affect ATP and the NAD+/NADH ratio, but also it can detrimentally affect the biosynthesis of different components such as neurotransmitters (Dienel, 2019) and hepatic sialic acid (Peng et al., 2023). Moreover, 2 NADH shuttles (pentose phosphate shunt, malate–aspartate) contributing to glycolysis and glycogen turnover would be affected by glucose deficiency (Dienel, 2019).

Impaired brain energy metabolism can be compensated by selected dietary approaches which result in an increase in plasma ketone bodies (KBs) from the catabolism of fatty acids (Phillips et al., 2021). Ketogenic diets, which have been effective in treating pediatric epilepsy (Barañano and Hartman, 2008), can facilitate brain energy function by inducing nutritional ketosis (Ota et al., 2016; Jensen et al., 2020; Ashton et al., 2021). Several studies have shown a positive association between various ketogenic diets and a better cognitive performance (Krikorian et al., 2012; Xu et al., 2020; Ferraris et al., 2021; Roy et al., 2021). For instance, it has been reported that 3 months medium chain triglycerides (MCT) intervention (17.3 g/day) can improve cognitive performance in mild to moderate AD patients (Xu et al., 2020). In another study, an average dosage of 25.2 g of MCT containing 99.3% caprylic acids, 0.6% capric acids, and 0.1% lauric acid for 4 months significantly improved the cognitive performance in AD patients (Juby et al., 2022). However, relatively few studies have reported the effects of this diet on AD-putative CSF and blood biomarkers such as Aβ, p-tau, GFAP, and NFL (Table 1). Moreover, there has been no human studies to date that have been conducted on the effects of ketogenic diet on these blood biomarkers. While recent studies indicate that ketogenic diet may impact AD biomarkers, it has not yet been determined whether increased KBs *per se* causes such changes or that KBs acts indirectly by increasing brain energy metabolism. Therefore, we aim to discuss how a ketogenic diet could provide an alternative energy source when glucose is not accessible. Furthermore, the influence of this diet on AD-associated biomarkers and other related risk factors is discussed. To the best of our knowledge, this is the first review article discussing recent studies on how different ketogenic diets or supplementations can affect these putative AD biomarkers and other AD related risk factors.

**Table 1:** Human, *in vitro* and animal studies on the association between ketogenic intervention and AD biomarkers.

Human studies					
Ketogenic Intervention	Dose, duration	Subjects	AD biomarker	Outcomes	Reference
MMKD (<10% carbohydrate, 60–65% fat, and 30–35% protein)	Daily MMKD for 6 weeks	11 MCI participants, (Age: 64.6 ± 6.4 yr)	CSF Aβ42, Aβ40, tau, and p-tau181	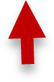 Aβ42, 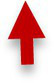 Aβ-42/40, 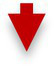 total tau, 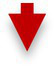 p-tau181	Nagpal et al. (2019)
MMKD (5–10% carbohydrate, 60–65% fat, and 30% protein)	Daily MMKD for 6 weeks	20 participants; MCI (*n* = 11) and SMC (*n* = 9), (Age: 64.6 ± 6.4 yr)	CSF Aβ42, Aβ40, t-tau, p-tau181, NFL	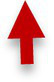 Aβ42, 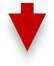 t-tau (only MCI), 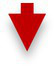 NFL, no changes in p-tau 181, Aβ40 or Aβ42/40	Neth et al. (2020)
kMCT (60% caprylic acid, 40% capric acid)	30 g/day kMCT for 6 months	MCI (*n* = 19), Aged ≥55 yr	IL-8, IL-6, IL-10, IL-17	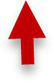 Circulating IL-8, No changes in IL-6, IL-10, IL-17	Myette-Côté et al. (2021)

*In vitro* studies				
Ketogenic Intervention	Dose, duration	AD biomarker	Outcomes	Reference
VCO	2 g of VCO for 24-48 h	Aβ40, Aβ42	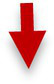 Aβ neurotoxicity, 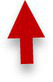 Neural survival	Nafar and Mearow (2014)
AcAc & βHB	Combination of 20 mM AcAc/20 mM βHB for 48 h	Aβ40	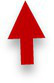 Aβ peptide efflux	Versele et al. (2020)

Animal studies						
Ketogenic intervention	Dose, duration	Pathology stage and age	Type of animal model	AD biomarker	Outcomes	References
βHB and AcAc	βHB: 600 mg/kg/day, AcAc: 150 mg/kg/day for 2 months	Early stage of AD at 4 months old	APP mouse model (*n* = 12–14)	Aβ42, Aβ40 and Aβ38	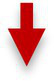 Accumulation of intracellular Aβ42. No changes in Aβ40 and Aβ38	Yin et al. (2016)
βHB	1.5 mmol/kg/d βHB for 28 days	Mid-stage of AD with cognitive deficit at 3.5 months old	APP/PS1 mice	Aβ oligomer, APP, IL-1β, TNF-α, and IL-6	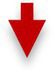 Aβ42 accumulation, 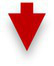 Senile plaques, APP expression, 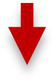 IL-1β, TNF-α, IL-6	Wu et al. (2019)
βHB	1.5 mmol/kg/day of βHB for 4 weeks	_	ApoE^−^ C57BL/6 J mice	Aβ, Tau	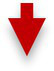 tau tangles, 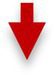 Aβ plaques	Krishnan et al. (2020)
KET (D-β-hydroxybutyrate and (R)-1,3-butanediol)	152 mg/dL for 300 days	Advanced stage of AD at 8.5 months old	3xTgAD mouse model	Aβ, pTau	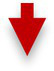 Aβ aggregation, 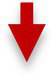 tau phosphorylation	Kashiwaya et al. (2013)
MCT diet (84% kcal fat, 13% kcal protein and 2% kcal carbohydrates)	MCT diet for 8 weeks.	High fat diet-induced cognitive impairment from 8 weeks old	Male C57BL/6 mice (*n* = 16)	NF-κB, TNF-α, APP and p-tau	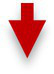 pTau/Tau ratio, 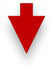 APP expression, 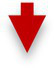 TNF-α, 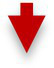 NF-κB,	Lin et al. (2022)
Ketogenic TD.96355 containing 90.5% fat, 0.3% carbohydrate, and 9.2% protein	KD for 30 days	TBI-induced neuronal loss at 6–7 weeks old	Male ICR mice (*n* = 26)	GFAP	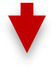 GFAP in dentate gyrus, 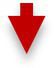 Reactive astrocytes.	Har-Even et al. (2021)
3-hydroxybutyl-3-hydroxybutyrate	0.5 mL/kg/day KE for 30 days	TBI-induced morphological and functional deficits at 8 weeks old	Male Sprague Dawley rats (*n* = 32)	GFAP	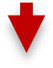 GFAP, 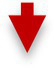 Brain astrogliosis, 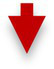 Neuroinflammation	Almeida-Suhett et al. (2022)
Ketogenic TD 96355	Daily for 30 days	Kainic acid-induced-hippocampal neuroinflammation	Male ICR mice (*n* = 30)	TNF-α, NF-κB, PPARγ	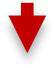 TNF-α levels, 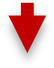 NF-κB, 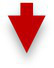 PPARγ	Jeong et al. (2011)
βHB	1.5 mmol/kg/d for 8 weeks	Chronic cerebral hypoperfusion-induced-neuroinflammation with cognitive impairment in adult rats	Male Sprague–Dawley rats (*n* = 25)	NF-κB, TNF-α, IL-1β	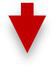 NF-κB, 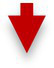 IL-1β, 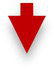 TNF-α	Wang et al. (2023)

MMKD, Modified Mediterranean Ketogenic Diet; MCI, Mild Cognitive Impairment; CSF, Cerebrospinal Fluid; Aβ, Amyloid Beta; p-tau, phosphorylated tau; SMC, Subjective memory complaints; NFL, Neurofilament Light; kMCT, ketogenic Medium Chain Triglycerides; IL, Interleukin; VCO, Virgin coconut oil; AcAc, Acetoacetate; βHB, βeta Hydroxybutyrate; APP, amyloid precursor protein; TNFα, Tumor Necrosis Factor alpha; ApoE, Apolipoprotein E; KET, Ketone esters; NF-κB, Nuclear factor kappa B; GFAP, Glial fibrillary acidic protein; PPARy, Peroxisome Proliferator-Activated Receptor y; PS1, Presenilin 1; TBI, Traumatic Brain Injury.

## 2. Ketogenic diet

Dietary approaches, with their holistic properties, have gained much attention over the last 4 decades (Mazzucca et al., 2021). Evidence has shown that dietary approaches can prevent or treat chronic diseases such as cardiovascular diseases, cancer, chronic respiratory diseases, and diabetes (Ojo, 2019). A ketogenic diet is a high-fat, moderate protein and low-carbohydrate diet primarily used for the treatment of drug-resistant epilepsy (Jiang et al., 2022). In this diet, the total calories are largely obtained from fat with protein and carbohydrates making a relatively lower contribution (D’Andrea Meira et al., 2019).

Based on the percentage of fat, carbohydrate and protein, ketogenic diets are categorized into different groups which include the following: Classic Ketogenic Diet (CKD), traditional MCT diet, modified MCT diet (Schwartz et al., 1989) and Modified Mediterranean Ketogenic Diet (MMKD) (Nagpal et al., 2019). CKD is typically comprised of 90% fat, 7% protein and 3% carbohydrate and is the most stringent ketogenic diet. Due to its anticonvulsant properties, CKD was used for treating epilepsy (Bough and Rho, 2007; Ferraris et al., 2021; Pietrzak et al., 2022). Two alternative forms of CKD are traditional MCT (60% MCT oil, 21% proteins and 19% carbohydrates) and modified MCT (30% MCT oils, 40% long chain saturated fat, 11% proteins and 19% carbohydrates) diets which consist of ketogenic kMCT (Schwartz et al., 1989; Neal et al., 2009). MMKD encompasses 60–65% fat, less than 10% carbohydrate, and 30–35% protein (Nagpal et al., 2019).

kMCT was first recognized by Huttenlocher in 1971 as a more tolerant and palatable form of CKD (Huttenlocher et al., 1971). MCT is a 6- to 12-chain-length carbon atom found abundant in coconut, palm kernel, and mammalian milk (Taylor et al., 2019; Mett and Müller, 2021). Based on their chain length, MCT is classified into hexanoic acid (caproic acid; C6), octanoic acid (caprylic acid; C8), decanoic acid (capric acid; C10), and dodecanoic acid (lauric acid; C12) (Nimbkar et al., 2022). Due to the higher capric/caprylic content with a higher potential to produce KBs, MMKD and kMCT have been suggested to have greater ketogenic properties than CKD (Huttenlocher, 1976; St-Pierre et al., 2019). Unlike longer chain fatty acids which require specific transporters such as CD36, fatty acid transport proteins (FATPs) and carnitine shuttle (Heidt et al., 2023), medium chain fatty acid (MCFA) arising from MCT do not rely on specific transporters to pass through the mitochondrial membrane (Miyagawa et al., 2018; Heidt et al., 2023). Through passive diffusion, MCFA can be easily and directly transported into the mitochondrial matrix (Heidt et al., 2023), and enhances beta-oxidation (β-oxidation) rate in hepatocytes, which ultimately increases the serum KB concentration (Ameen et al., 2022). In response to the increased levels of KBs, brain energy metabolism and cognitive functions are improved significantly (Ameen et al., 2022). Increased levels of KB and brain energy might be important especially for the treatment of patients with AD, impaired brain energy metabolism and cognitive dysfunction (Krikorian et al., 2012).

## 3. Ketogenic intervention: metabolic pathways from oxidation to ketolysis

**Figure 1:**
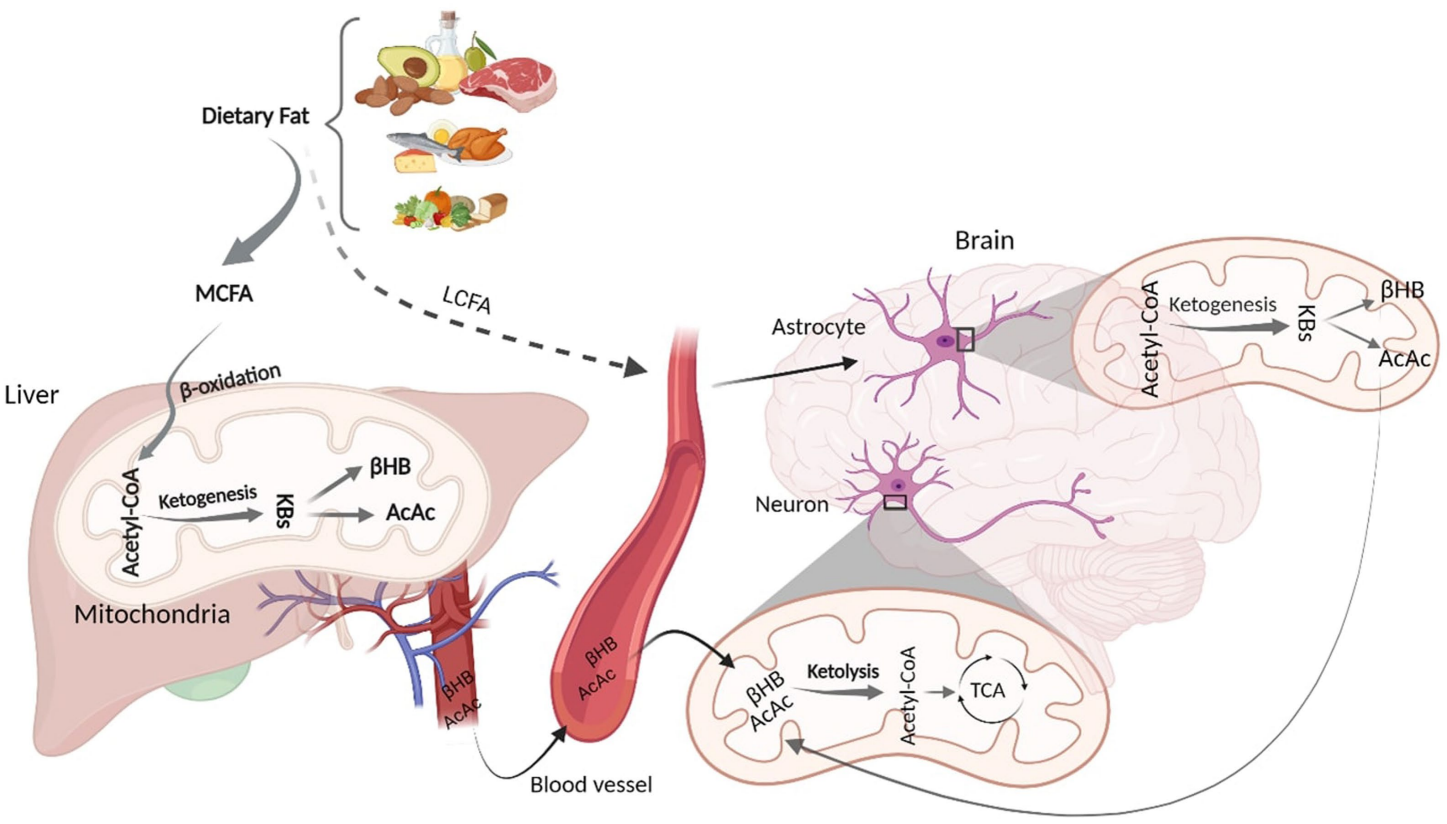
Schematic figure of Ketone Body Synthesis and Metabolism. Dietary MCT is hydrolysed into MCFA, which undergoes β-oxidation to produce acetyl-CoA. In the liver mitochondria, excessive acetyl-CoA induces ketogenesis and produces KBs (βHB and AcAc). Via circulation, KBs enter the brain and inside the neural mitochondria, they induce ketolysis to produce acetyl-CoA. LCFAs that bypass liver metabolism goes to the astrocytes and initiate β-oxidation, generating acetyl-CoA that undergoes ketogenesis to provide surplus KBs, which go to the neurons to furnish further energy. KD, Ketogenic Diet; MCFA, Medium Chain Fatty Acid; KBs, Ketone Bodies; βHB, βeta Hydroxybutyrate; AcAc, Acetoacetate; TCA, Tricarboxylic Acid. Adapted from Biorender.com.

Although glucose is a primary energy source for the brain, KBs provide up to 60% of brain energy during glucose restrictions (Pietrzak et al., 2022). MCT ketogenic diet mimics fasting-associated metabolisms, during which glucose is replaced with fatty acids (Augustin et al., 2018; Włodarek, 2019). Similar to fasting, MCT- and any kind of ketone-rich diet induce nutritional ketosis (Włodarek, 2019). This is generally characterized by elevated concentration of KBs from a normal range (~0.5 mM) to higher levels (3 mM) which considers an optimal range of KBs in serum (Harvey et al., 2019). This increased level of serum KBs is achieved via shortage of carbohydrates (Harvey et al., 2019). The metabolic pathway of ketone bodies from synthesis in liver and astrocytes to energy generation in the brain is shown in Figure 1. Shortly after the consumption of a ketogenic meal, MCT is hydrolyzed into MCFA. Via portal circulation, MCFAs are transported into the liver and in the hepatocytes, they undergo β-oxidation, which are converted to acetyl-CoA, thereby initiating ketogenesis to yield KBs. Three types of KBs, including beta-hydroxybutyrate (βHB), acetoacetate (AcAc), and acetone, are generated from the hydrolyzation of fatty acids (Watanabe et al., 2020). These KBs are produced in the liver as the primary site of ketone synthesis, albeit they cannot be utilized by hepatocytes as the liver does not have ketolysis-related enzymes (enzyme Oxct1/SCOT1) to metabolize Acetoacetyl-CoA (AcAc-CoA) (Bendridi et al., 2022). The newly synthesized KBs flow to the extra-hepatic tissues, including brain (Bendridi et al., 2022). As their levels increase in the bloodstream, KBs enter the brain via monocarboxylate transporters located in Blood Brain Barriers (BBB) (Jensen et al., 2020). KBs are delivered to neurons via different types of monocarboxylate transporters localized in astrocytes and neurons. Monocarboxylate transporters 1, 3 and 4 expressed in astrocytes and monocarboxylate transporter 2 expressed in neurons are responsible for KB transportations (Ardanaz et al., 2022). However, the expression of these fundamental transporters, as the brain bioenergetic carriers, is reduced in AD pathology (Ding et al., 2020). In the healthy individuals, after KBs were transported into the neurons, inside the mitochondria, they induce ketolysis. KBs are then converted to Acetyl-CoA, which enters the tricarboxylic acid cycle to produce energy for the neurons (Jensen et al., 2020).

Although hepatocytes are the primary site of KBs production, to a lesser extent, other extrahepatic tissues, such as astrocytes, have the capacity to produce KBs from some fatty acids (long-chain; >12 carbons) (Takahashi, 2020). Both *in vitro* and animal studies have demonstrated that astrocytes can synthesize KBs due to their ability to oxidize fatty acids (Yudkoff et al., 1997; Yoon and Jo, 2012). It has been found that some fatty acids can bypass liver metabolism and undergo ketogenesis in astrocytes (Yudkoff et al., 1997; Yoon and Jo, 2012; Nonaka et al., 2016). Astrocytes are the main site of mitochondrial β-oxidation and are the only source of KB generation in the brain (Yang et al., 2022). Fatty acids, transported to the astrocytes, can initiate β-oxidation to produce acetyl-CoA. The produced acetyl-CoA undergoes ketogenesis to provide surplus KBs (Nonaka et al., 2016; Silva et al., 2022), which then moves to the neighboring neurons via monocarboxylate transporters. In the neurons, KBs undergo ketolysis to produce Acetyl-CoA to make further energy fuels for the brain (Nonaka et al., 2016; Jensen et al., 2020; Takahashi, 2020). This can ameliorate the energy crisis in the brain when glucose is not accessible (Poff et al., 2021). Therefore, an adequate and continuous brain energy supply provided by KBs can repair the brain metabolism (Jensen et al., 2020). In addition to improved brain energy metabolism, ketogenic diets are associated with alteration in AD CSF biomarkers (Neth et al., 2020). However, the mechanism of action of ketogenic diets in altering AD biomarker levels remains to be elucidated.

## 4. Effects of the ketogenic diet on multiple AD biomarkers

The beneficial effects of ketogenic diets on cognitive performance have been reported widely in healthy individuals as well as those with mild, moderate, and severe AD (Fortier et al., 2019, 2021; Xu et al., 2020; Yomogida et al., 2021; Juby et al., 2022). In two human studies, 30 g/day of kMCT containing 12% Captex 355 (60% caprylic acid, 40% capric acid) mixed with lactose-free skim milk improved executive function (Fortier et al., 2019, 2021). An improvement in episodic memory, processing speed and language has been reported in participants with MCI after a 6-month treatment with kMCT (Fortier et al., 2019, 2021). An average dosage of 25.2 g of MCT containing 99.3% caprylic acids, 0.6% capric acids, and 0.1% lauric acid for 4 months significantly improved the cognitive performance in AD patients (Juby et al., 2022).

Daily consumption of a jelly preparation containing 17.3 g MCT within 3 months showed improved cognitive ability in mild to moderate AD patients (Xu et al., 2020). Meiji817-B is a MCT meal containing ketogenic milk with 30.3 g caprylic acid and 9.8 g capric acid per 100 g total fat (Yomogida et al., 2021). Meiji817-B exhibited improved executive function, including working memory or inhibitory control in healthy elderly subjects (Yomogida et al., 2021). These cognitive benefits were positively associated across various ketogenic diets including MCT oil, MCT powder and MCT jelly (Fortier et al., 2019, 2021; Xu et al., 2020; Yomogida et al., 2021; Juby et al., 2022). However, there is a paucity of information on the effects of this diet on AD CSF or plasma biomarkers (Table 1). Therefore, herein, we aim to review the novel studies on the effects of ketogenic diet on several AD fluid biomarkers such as Aβ, tau, GFAP, and NFL as well as to explore their involvement in other AD related risk factors.

### 4.1. Effect of ketogenic diet on Aβ biomarkers

Amyloid plaque, which is the extracellular abnormal deposition of Aβ, is a major neuropathological hallmark of AD (Murpy and LeVine III, 2010; Qiu et al., 2015; Moore et al., 2018; Tarawneh, 2020). Increased brain Aβ load along with reduced concentration of Aβ in the CSF and plasma have been widely reported (Hansson et al., 2019; Zaretsky et al., 2022; Chatterjee et al., 2023).

**Figure 2:**
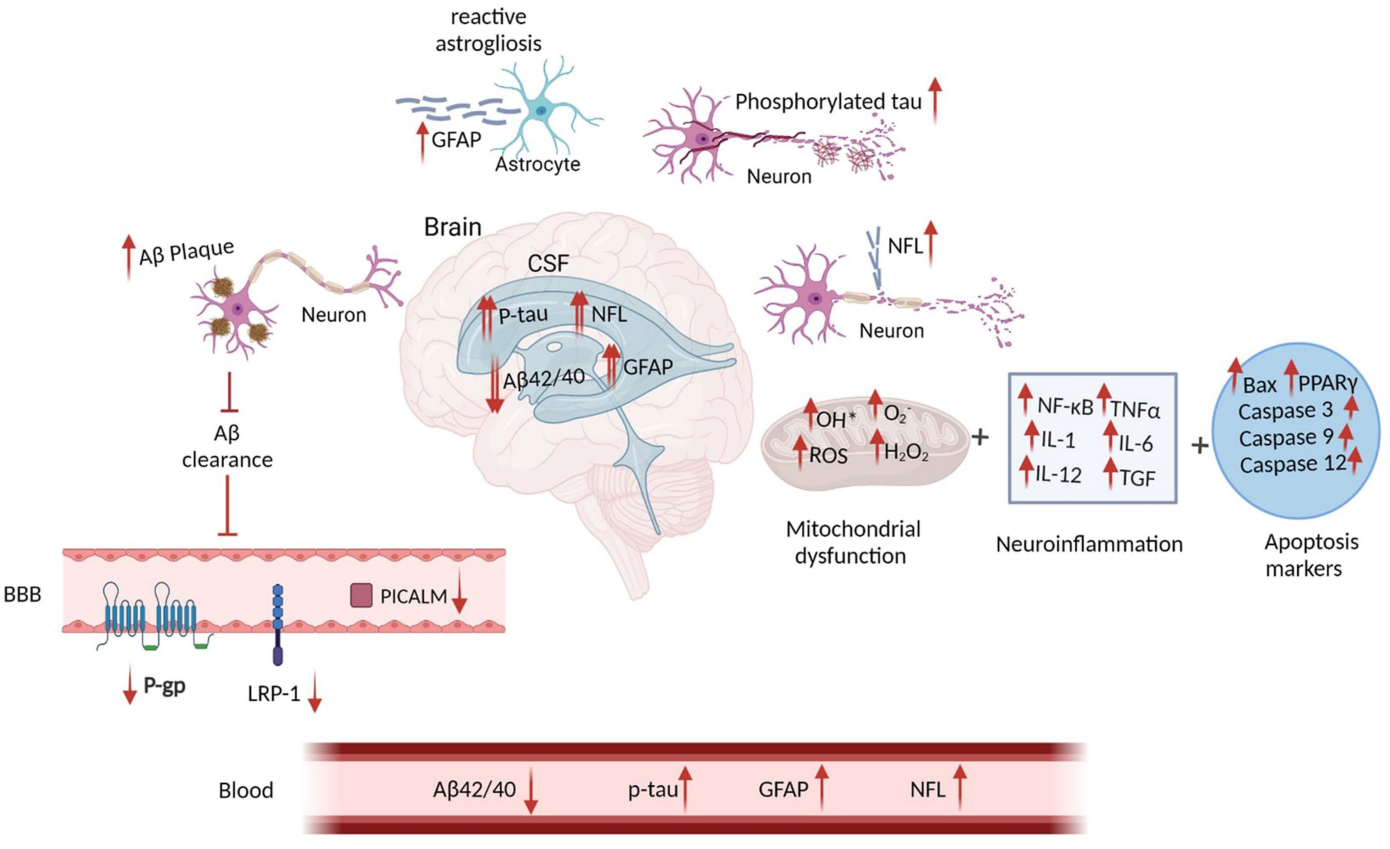
The Schematic Figure of Various Pathological Status of AD Biomarkers in the Fluid. Increased Aβ plaques inside the brain suppress the Aβ clearance and reduce Aβ efflux. This mitigates 3 Aβ transporters including LRP1, RAGE and P-gp within the BBB. Aβ deposition in the brain leads to reduced CSF and plasma Aβ. Injured neurons increase the levels of CSF and blood p-tau. Reactive astrogliosis increases GFAP levels in both CSF and blood. Axonal neural damage also releases higher NFL. Although disruption of these biomarkers can be found in both CSF and blood, their concentrations are significantly higher in the CSF (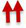) rather than on blood (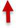). In addition, Mitochondrial deficits, neuroinflammation and apoptosis are all closely linked to the AD pathology. Aβ, Amyloid Beta; GFAP, Glial fibrillary acidic protein; NFL, Neurofilament Light; ROS, Reactive Oxygen Species; NF-κB, Nuclear factor kappa B; TNFα, Tumor Necrosis Factor alpha; IL, Interleukin; TGF, Transforming Growth Factor; BAX, Bcl-2-associated X; PPAR, Peroxisome Proliferator-Activated Receptor; P-gp, P-glycoprotein; LRP1, lipoprotein receptor-related protein-1; PICALM, Phosphatidylinositol-binding clathrin assembly protein; BBB, Blood Brain Barrier. Adapted from Biorender.com.

**Figure 3:**
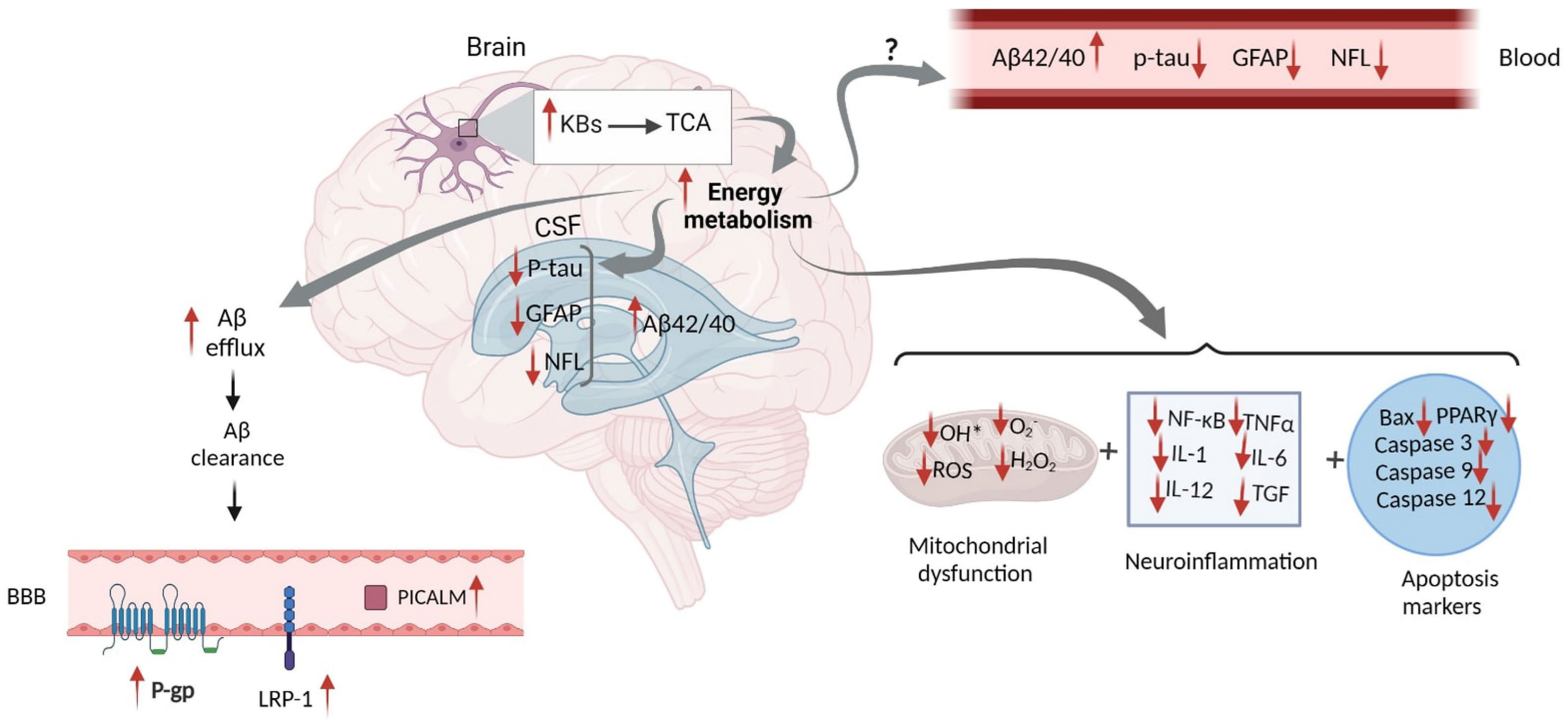
Proposed Schematic Figure for the Effects of Ketogenic Intervention on Different AD Related Markers in the CSF and Blood. KBs in the neural mitochondrial go to the TCA cycle, which increases energy metabolism. This increases Aβ efflux and facilitates Aβ clearance in the brain. Higher Aβ clearance across the BBB leads to the elevation of LRP-1, P-gp, and PICALM proteins, which altogether result in the increased concentration of Aβ in the CSF. Increased energy metabolism might reduce tau hyperphosphorylation, GFAP expression, and NFL release in the brain. However, the mechanism by which KBs can eliminate their levels in the CSF and blood has not yet been discovered. In addition, increased energy metabolism might actively impact mitochondrial function and reduce neuroinflammation and apoptosis markers. Aβ, Amyloid Beta; GFAP, Glial fibrillary acidic protein; NFL, Neurofilament Light; ROS, Reactive Oxygen Species; NF-κB, Nuclear factor kappa B; TNFα, Tumor Necrosis Factor alpha; IL, Interleukin; TGF, Transforming Growth Factor; BAX, Bcl-2-associated X; PPAR, Peroxisome Proliferator-Activated Receptor; P-gp, P-glycoprotein; LRP1, lipoprotein receptor-related protein-1; PICALM, Phosphatidylinositol-binding clathrin assembly protein; BBB, Blood Brain Barrier. Adapted from Biorender.com.

BBB is essential in maintaining Aβ metabolism, and any abnormalities in BBB might impair Aβ normal transport, thereby causing Aβ accumulation and deposition (Wang et al., 2021). It has been demonstrated that the expression of different Aβ transporters in BBB is decreased in the AD brain (Wang et al., 2021). Three major proteins, including low-density lipoprotein receptor-related protein 1 (LRP1), AΒCB1 as P-glycoprotein (P-gp), and phosphatidylinositol-binding clathrin assembly protein (PICALM) have a pivotal role in the efflux of Aβ peptides across BBB (Storck et al., 2018). Reduced LRP1 and P-gp, as the major Aβ transporters across the BBB, further contributes to the poor clearance of brain Aβ (Figure 2). In contrast, increased KBs can facilitate the efflux of Aβ peptides across a human *in vitro* BBB *model* by enhancing LRP1, PICALM, and p-gp (Figure 3). This improves the Aβ transportation and clearance resulting in less Aβ plaque deposition and slower release of soluble Aβ (Versele et al., 2020).

Increased Aβ efflux reduces Aβ plaque deposition and mitigates soluble Aβ (Wang et al., 2021). Reduced levels of soluble oligomer Aβ42 was reported followed by the combined treatment of AcAc and βHB (Yin et al., 2016). An *in vitro* study showed a significant decline in neurotoxicity due to lowered Aβ plaque deposition and reduced soluble Aβ after consuming coconut oil (CoOil) (Nafar and Mearow, 2014). βHB therapy (1.5 mmol/kg/d) in AD model of mice for 28 days suppressed APP expression, enhanced the expression levels of neprilysin, as a degradation enzyme for Aβ, reduced number of senile amyloid plaques, and mitigated soluble and insoluble Aβ42 and Aβ40 (Wu et al., 2019).

Increased oxidative stress and changes in brain energy availability are associated with impaired ATP-sensitive potassium (K_ATP_) channels, found in both glia and neurons. K_ATP_ channels known as metabolic sensors are changed across the AD continuum (Grizzanti et al., 2022). Activation of Kir6.2, one of the main subunits of K_ATP_ channels, is increased as Aβ pathology is elevated (Grizzanti et al., 2022). In an animal study, APP/PSE1 mice knocking out Kir6.2, showed no significant increase in Aβ pathology, while activation of K_ATP_ channel showed Aβ deposition (Grizzanti et al., 2023). On the other hand, in an *in vitro* study, a ketone cocktail (BHB and AcAc; each 1 mM) treatment through their interactions with K_ATP_ channels guaranteed neural survival, increase ATP production (Kim et al., 2015; Pietrzak et al., 2022) and decrease Aβ aggregations (Pietrzak et al., 2022).

In a human trial, a 6-week MMKD intervention containing 60–65% fat, 30% protein and 5–10% carbohydrate potentially increased CSF Aβ42 and Aβ42/tau ratio in the at-risk adults compared to control groups who consumed 55–65% carbohydrate, 15–20% fat, and 20–30% protein (Neth et al., 2020). Likewise, in another human study, compared to cognitively unimpaired participants, MCT-treated participants showed a significantly increase CSF Aβ40 and Aβ42 over a six-week MMKD therapy containing >10% carbohydrate, 60–65% fat, and 30–35% (Nagpal et al., 2019). Accordingly, it is suggested that KBs arising from various types of ketogenic interventions can decrease Aβ neurotoxicity, reduce Aβ aggregation and modulate Aβ peptides in the circulation. Ketone molecules are also able to increase Aβ efflux and mitigate soluble Aβ.

### 4.2. Effect of ketogenic diet on tau biomarkers

Tau protein, a core hallmark of AD, is the main constituent of the paired helical filaments (PHF), which forms neurofibrillary tangles (NFTs) in the AD brain (Serrano-Pozo et al., 2011). Increased CSF and plasma phosphorylated tau, commences decades before the clinical presentation of the disease (Rajmohan and Reddy, 2017; Chatterjee et al., 2022). However, the neuroprotective antioxidant properties of compounds present in some ketogenic diets can ameliorate abnormal tau aggregation and induce neural survival (Guo et al., 2013; Baek et al., 2020; Krishnan et al., 2020).

There are few evidence supporting neuroprotective features induced by ketogenic diet on tau biomarkers, though some animal studies and only 2 human studies were conducted to show their association. For example, 4 weeks βHB therapy, in the C57BL/6 mice models of ApoE-deficient AD, which causes progressive p-tau Ser^202^/Thr^205^ accumulation, significantly ameliorated tau tangles colocalized in the hippocampal region of ApoE4 transgenic mice (Krishnan et al., 2020). This significantly reduced the risk of AD progression in these mice (Krishnan et al., 2020). Increased number of intracellular p-tau in the amygdala, subiculum, CA1 and CA3 of the hippocampus in AD male 3xTgAD mice models has been modified followed by taking a ketone ester diet comprising of D-β-hydroxybutyrate and (R)-1,3-butanediol (Kashiwaya et al., 2013). Prolonged consumption (16 weeks) of a high-fat-high cholesterol diet in C57BL/6 mice model of AD showed hyperphosphorylation of p-tau S396 and increased neuroinflammation in cortex and hippocampus (Lin et al., 2022). Albeit, an eight-week treatment of MCT diet composed of 84% fat, 2% carbohydrates and 13% protein significantly reduced the ratio of p-tau S396 /total-tau (t-tau) in these regions (Lin et al., 2022). MCT mitigated the hyperphosphorylation of p-tau S396 and reduced neuroinflammation (Lin et al., 2022).

A decline in CSF t-tau concentration was found in the MCI subjects after a 6-week consumption of MMKD (Neth et al., 2020). However, the same study showed no significant changes in the levels of p-tau181 in the subjective memory complainer (SMC) group (Neth et al., 2020). Compared to American Heart Association Diet (AHAD), a low-fat and higher-carbohydrate diet, MMKD over a 6-week therapy significantly impeded CSF t-tau in MCI or SMC patients (Nagpal et al., 2019). Therefore, it can be suggested that the levels of tau in some isoforms is reduced followed by increased levels of KBs in the circulation.

### 4.3. Effect of ketogenic diet on astroglial biomarkers with a focus on GFAP

AD pathogenesis is not exclusively limited to the formation of Aβ plaque and tau phosphorylation. It includes some other factors that contribute to neuropathological processes (Pereira et al., 2021). Astroglial-dependent toxicity plays a leading role in the development of AD pathology. Astrogliopathy refers to hyperactivation, astroglial atrophy and loss of function due to the destruction of adjacent neurons (Verkhratsky et al., 2019). In AD, reactive astrocytes acquire neurotoxicity arising from astrocyte hypertrophy (Perez-Nievas and Serrano-Pozo, 2018), which can provide an anatomical substrate for the aberrant growth of newborn dentate granule cells (Robinson et al., 2016). Intermediate filament (IF) cytoskeleton changes lead to the overexpression of IF proteins such as GFAP, an index for astroglia activation, which gradually increases followed by any neurodegenerative injuries (Smit et al., 2021). Astrogliopathy can affect the level of biomarkers and can be found in the early stages of AD (Verkhratsky et al., 2019; Pereira et al., 2021). Astrocytic phagocytosis also mediates the Aβ clearance in the brain through the influx and degeneration of soluble form of Aβ (Frost and Li, 2017). It has been reported that even with a slight deficiency or decline in Aβ clearance, neurotoxicity occurs, which is correlated to a higher risk of AD (Yoon and Jo, 2012). In astrogliosis, the levels of some biomarkers such as GFAP (Chatterjee et al., 2021) and vimentin (Dai et al., 2023) are elevated significantly.

Higher levels of GFAP in the hippocampus and cortex have been reported caused by neural loss, cognitive and memory deficits in a TBI mouse model (Har-Even et al., 2021). However, a 30-day treatment of ketogenic diet (90.5% fat, 9.2% protein, and 0.3% carbohydrate) attenuated neural loss, and improve memory function through mitigating reactive astrocytes. GFAP concentration in the dentate gyrus but not in the cortex was significantly reduced, followed by an increase in blood KBs levels (Har-Even et al., 2021).

Increased levels of βHB significantly reduced the hyperactivation of astrocytes and three other hyperactivated microglial markers, including ionized calcium-binding adaptor molecule 1 (Iba-1), a M1 microglial marker (CD16/32), and a marker of macroglia (CD68) (Zhang et al., 2020). Higher levels of GFAP in response to regional astrogliosis in the mouse hypothalamus and the hippocampal network is associated with memory decline and further neural damage (Bondan et al., 2019). However, by producing KBs, ketone monoester and 3-hydroxy butyl-3-hydroxybutyrate provides an alternative brain fuel, which compensates for the reduction in glucose utilization, and combats astrogliosis, and microgliosis in the prefrontal cortex, cortex, amygdala, and hippocampus (Le Foll and Levin, 2016; Morris et al., 2020; Almeida-Suhett et al., 2022). Thirty days treatment with 3-hydroxybutyl-3-hydroxybutyrate (0.5 mL/kg/day) were able to reduce prominent GFAP positive reactive astrocytes in the TBI-induced behavioral and neuropathological alterations (Almeida-Suhett et al., 2022). The neuroprotective mechanism of KBs is a possible mechanism that ultimately changes the levels of GFAP (Figure 3).

It has been shown that 8 weeks intervention therapy with MCT composed of 84% fat, 2% carbohydrates, and 13% protein could significantly decrease GFAP expression in the cortical and hippocampal regions of the C57BL/6 mice brain (Lin et al., 2022). This effect was ascribed to the presence of caprylic acid and capric acid in the MCT diet, which significantly elevated βHB in the circulation (Lin et al., 2022). Therefore, it is suggested that βHB or combined KBs arising from different types of KDs mitigate reactive astrogliosis and reduce the levels of GFAP in circulation.

### 4.4. Effect of ketogenic diet on NFL

NFL, a neuronal cytoplasmic biomarker, has emerged as one of the most promising candidates for the diagnosis and progression of neurodegenerative disorder (Dhiman et al., 2020). In neurological disorders including inflammatory, neurodegenerative, traumatic, and vascular diseases, the release of this biomarker is highly increased in response to severe axonal damage (Gaetani et al., 2019). Elevated NFL levels correlate with poorer cognitive performance, brain hypometabolism, and atrophy, making this biomarker a promising candidate for detecting neurodegeneration and AD (Mattsson et al., 2017).

Little is known about the association between ketogenic intervention and changes in NFL concentrations. The modified ketogenic diet may impact axonal neural injuries (Neth et al., 2020). Six-week treatment with MMKD could significantly reduce CSF NFL biomarkers in the older populations with MCI, suggesting ketogenic intervention by inducing ketosis can constructively impact neurodegeneration-related injuries. While only one human study has been conducted on the impact of KDs on NFL levels (Neth et al., 2020), it is proposed that KBs can mitigate NFL levels in the circulation.

### 4.5. Effect of ketogenic diet on neurotrophic, neuroinflammation, apoptotic and oxidative stress factors

In addition to aforementioned biomarkers with prognostic and diagnostic value, AD is characterized by increased multiple metabolic interactions and comorbidities that promote its progression (Karikari et al., 2020; Verberk et al., 2022). As the disease progresses, AD patients exhibit further abnormalities, such as down regulation of neurotrophic factors including brain-derived neurotrophic factor (BDNF) through dysregulation of the glutamatergic *N*-methyl-D-aspartate receptor (NMDAR) which can cause Aβ-induced neuronal loss and dendritic atrophy (Meng et al., 2013). Downregulation of BDNF as a potential diagnostic biomarker is manifested during prodromal stage to severe AD (Bessi et al., 2020). On the contrary, 14 days injection of 100 mg/kg AcAc showed a higher expression of hippocampal BDNF in the mice model of familial AD (Wu et al., 2022), which is assumed to be due to neuroprotective properties induced by AcAc (Murugan and Boison, 2020; Zhang et al., 2023). Expression of BDNF increased followed by 2 weeks intermittent fasting in the mouse models of Parkinson disease (Ojha et al., 2023). In addition, in a human study on 15 healthy subjects, combined caprylic acid (20 g) and coconut oil (30 g) after 4 h significantly increased serum levels of precursor BDNF, but not mature BDNF (Norgren et al., 2021).

In 2004, the mitochondrial cascade hypothesis was reported, describing it as a prerequisite that leads to disease progression in AD (Swerdlow and Khan, 2004). The percentage of mitochondrial dysfunction and depolarization is increased with age which ultimately increases the level of free radicals and insoluble Aβ from APP (Swerdlow and Khan, 2004). Increased levels of free radicals, such as Reactive Oxygen Species (ROS) and hydrogen peroxide (H_2_O_2_) are associated with cellular oxidative damage and disruption of cellular integrity (Agrawal and Jha, 2020). One of the main events after increased oxidative stress is DNA damage and consequently cell death which is considered to be one of the main events in neurodegeneration (Shadfar et al., 2022, 2023). However, the effects of ketogenic diet on oxidative stress and DNA damage in AD models have not yet been investigated.

It has been revealed that through reducing excessive levels of H_2_O_2_-induced neural injuries, MCFA capric acid can significantly suppress intracellular oxidative stress in the neuroblastoma cell line (Mett and Müller, 2021). Capric acid significantly inhibits the natural release of cellular H_2_O_2_ and reduces ROS levels (Mett and Müller, 2021).

In addition to mitochondrial dysfunction and surplus ROS production, several apoptosis-associated mediators such as p38, p21, mitogen-activated protein kinase (MAPK), and caspase 2, 3 and 9 can worsen the pathology of disease (Obulesu and Lakshmi, 2014; Misrani et al., 2021). Inversely, βHB can modulate neural apoptosis induced by low glucose accessibility due to mitochondrial dysfunction (Lin et al., 2022). βHB not only provides an alternative energy fuel for the brain, but also modulates cellular signaling transduction related to apoptotic pathogenesis (Lin et al., 2022). The antiapoptotic feature of βHB decreased apoptosis-related proteins, including p38 and caspase 3 and increase p-ERK (Lin et al., 2022). Exogenous βHB can suppress overexpression of p53, caspase-3, caspase-9, and caspase-12 in Aβ-induced cell apoptosis in the hippocampal network (Xie et al., 2015).

Pro-inflammatory mediators are other major markers for AD progression (Rajesh and Kanneganti, 2022), tightly coupled with oxidative stress (Agrawal and Jha, 2020). Increased nuclear factor kappa-B (NF-κB), interleukin-1 (IL-1), IL-6, IL-12, transforming growth factor beta (TGFβ), and tumor necrosis factor alpha (TNFα) increase the risk of AD (Su et al., 2016). However, treatment with ketogenic Harlan Teklad TD 96355 diet containing 90.5% fat, 0.3% carbohydrate, and 9.2% protein reduced hippocampal TNF-α and PPARγ activation and down regulate the expression of hippocampal COX-2. Reduced level of these markers results in the reduction of neurotoxicity and neuroinflammation (Jeong et al., 2011). Due to their anti-inflammatory properties, KBs can reduce the expression of pro-inflammatory factors IL-6 (Platero et al., 2020), TNF-α and IL-1β (Wu et al., 2019; Wang et al., 2023). In a human study, 6 months of kMCT drink in MCI patients could significantly increase circulating IL-8 levels with minor side effects (Myette-Côté et al., 2021). The nod-like receptor pyrin domain expression levels containing 3 (NLRP3) inflammasome and pro-inflammatory cytokines such as IL- 1β and TNF-α were significantly reduced after ketone therapy (Zhang et al., 2020). Reduced levels of NLRP3 could inhibit caspase-1 activation and pro-inflammatory pathways and suppress NF-κB which lead to neural survival (Gough et al., 2021). According to these studies, KBs can modulate inflammatory cytokines, reduce free radicals and apoptosis.

## 5. Research gaps, limitations, and future directions

Ketone bodies are known to be the main energy source for the brain when glucose is restricted. To date, many studies have examined the effects of ketogenic diet on cognitive function and glucose metabolism from the early preclinical stages to severe AD. However, there are major gaps in the existing literature that need to be addressed. Since a wide variety of intervention dose and duration has been reported, it is crucial to find out the most optimal dose and duration for ketogenic intervention with no/few side effects and maximum tolerability. To improve cognitive outcomes in AD, long-term adherence to ketogenic diet is imperative, therefore identifying a tolerable ketogenic diet that causes limited or few side effects will reduce dropouts and allow for a longer-term adherence.

Secondly, differing fatty acids stimulate differing levels of ketone body production. For instance, compared to LCFAs, MCFAs stimulate greater levels of ketone body production. It is hypothesized that brain energy resulting from increased ketone body response may delay AD progression and may subsequently result in a better outcome for AD biomarkers. So, as different ketogenic diets can provide differing ratios of fatty acids, it is important to consider the composition of fatty acids in the ketogenic diet in future studies.

While studies have shown improvements in cognition and brain energy metabolism following consumption of ketogenic intervention, few studies have reported the impact of ketogenic diets on AD biomarkers. Recent evidence has shown that AD biomarkers are capable of diagnosing disease 15 to 20 years prior to clinical onset. Therefore, more robust clinical studies are needed to investigate the effect of ketogenic diet on AD biomarkers to evaluate its effectiveness as a therapeutic approach to delay AD progression.

Lastly, the exact molecular mechanism on how KBs can make these changes has not yet been determined. As such, future studies are required to investigate whether KBs themselves directly alter the levels of biomarkers or whether it is mediated by its action to alter brain energy metabolism. Therefore, investigation of the mechanisms behind such possible changes should be a research priority for future clinical trials.

## 6. Conclusion

The current review of the clinical trials undertaken to date indicates that the majority of ketogenic dietary interventions induce ketosis to produce KBs which ultimately lead to the improvements in cognition. The KBs generated as a result of these interventions provide essential energy for the brain and thereby help to retard neurodegeneration, though more convincing evidence is needed. In addition to changes in brain energy metabolism, KBs can modulate fluid biomarkers associated with AD pathology. Animal, *in vitro* and some human studies have demonstrated that ketogenic intervention not only modulates AD putative biomarkers, such as Aβ 42/40, p-tau, GFAP and NFL, but also plays an effective role in the improvement of oxidative stress, inflammation, and mitochondrial mechanism. However, blood biomarkers while having the potential as both diagnostic and prognostic markers need considerably more investigation before their significance and contribution to brain health can be determined.

## Author contributions

MR: Writing – original draft, Investigation, writing review and editing. MF: Writing – review & editing, Investigation. SE: Writing – review & editing, Investigation. PA: Writing – review & editing. SS: Writing – review & editing. EB: Writing – review & editing, Investigation. HH: Writing – review & editing. SM: Writing – review & editing. MS: Writing – review & editing. PC: Conceptualization, Writing – original draft, Investigation, and Visualization. RT: Writing – review & editing, Investigation and Visualization. CD: Writing – review & editing. MG: Writing – review & editing, Investigation, Conceptualization and Visualization. RM: Writing – review & editing, Conceptualization, Project administration, Supervision.
